# A standardized workflow for submitting data to the Minimum Information about a Biosynthetic Gene cluster (MIBiG) repository: prospects for research-based educational experiences

**DOI:** 10.1186/s40793-018-0318-y

**Published:** 2018-07-11

**Authors:** Samuel C. Epstein, Louise K. Charkoudian, Marnix H. Medema

**Affiliations:** 1 Department of Chemistry, Haverford College, Haverford, PA 19041-1391 USA; 2 Bioinformatics Group, Wageningen University, Droevendaalsesteeg 1, 6708PB Wageningen, The Netherlands

**Keywords:** MIBiG, Specialized metabolism, Biosynthetic gene cluster, Natural product, Course-based undergraduate research experience

## Abstract

Microorganisms utilize complex enzymatic pathways to biosynthesize structurally complex and pharmacologically relevant molecules. These pathways are encoded by gene clusters and are found in a diverse set of organisms. The Minimum Information about a Biosynthetic Gene cluster repository facilitates standardized and centralized storage of experimental data on these gene clusters and their molecular products, by utilizing user-submitted data to translate scientific discoveries into a format that can be analyzed computationally. This accelerates the processes of connecting genes to chemical structures, understanding biosynthetic gene clusters in the context of environmental diversity, and performing computer-assisted design of synthetic gene clusters. Here, we present a Standard Operating Procedure, Excel templates, a tutorial video, and a collection of relevant review literature to support scientists in their efforts to submit data into MiBIG. Further, we provide tools to integrate gene cluster annotation projects into the classroom environment, including workflows and assessment materials.

**Electronic supplementary material:**

The online version of this article (10.1186/s40793-018-0318-y) contains supplementary material, which is available to authorized users.

## Introduction

Biosynthetic gene clusters within microorganisms encode highly evolved molecular machines that catalyze the production of structurally complex specialized metabolites, many of which have been repurposed as pharmaceutical, agricultural, and manufacturing agents. Recent advances in genome sequencing have led to a flood of data about these BGCs, but how this information is reported, and where it is deposited, was inconsistent until recent efforts to create a standard systematic deposition procedure [1]. The Minimum Information about a Biosynthetic Gene cluster specification provides a robust community standard for annotations and metadata on biosynthetic gene clusters and their molecular products [1]. Additionally, the MIBiG repository [2] provides a centralized and global platform to store these standardized annotations. Scientists can submit new gene clusters to this repository through its online submission system. In a call to action, over 154 researchers joined forces to annotate a significant portion of the experimental data on hundreds of BGCs that have been characterized in recent decades [1]. These researchers also committed to submitting MIBiG-compliant data sets when publishing new experimental results on BGCs.

**Fig. 1:**
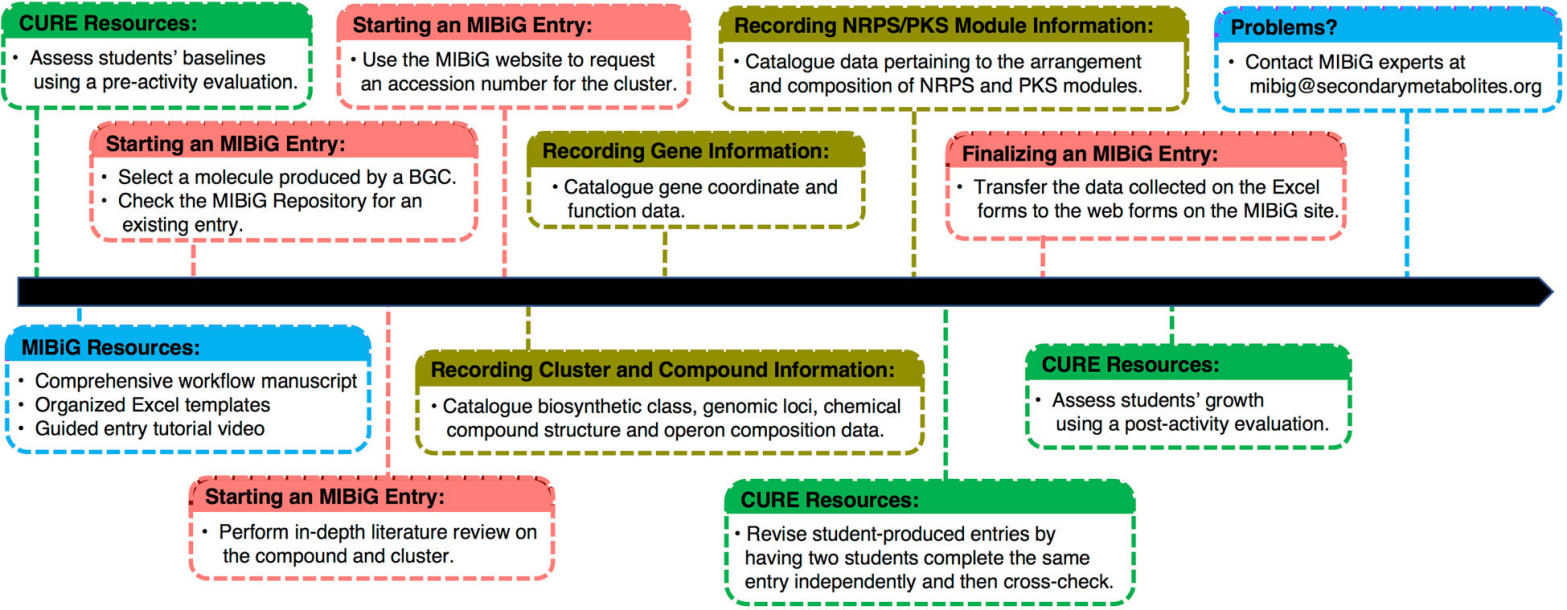
Pictorial organization of the typical order of operations during completion of MIBiG submissions. This figure illustrates how all the resources we provide here can be used alongside one another most effectively. The red and yellow boxes refer to logistical and data entry steps, respectively. The resources aimed to student annotation efforts in course-based undergraduate research experiences (CUREs) are shown in green. Lastly, resources aimed at mitigating potential user complications in filling out the database are shown in purple

To facilitate future depositions of BGC information into MIBiG, we herein present a detailed workflow, Excel templates that scaffold the annotation procedures, and a video tutorial in which the entire annotation process is presented for a sample BGC entry (Fig. 1). We envision that these resources will be of interest to research groups reporting new information on BGCs as well as faculty seeking to incorporate original research opportunities into pedagogical practices by bringing this bioinformatics-based challenge to the classroom. For the latter, the ~ 800 partially annotated BGCs in the MIBiG repository represent fertile ground for undergraduates to make meaningful contributions to the biochemistry community while developing their skills in scientific literacy and research [3].

## Standard operating procedure

### Overview

To properly catalogue the information on a specific BGC, one must be informed of the scope of experiments that have been performed on the biosynthetic pathway in question. Using credible sources, namely trusted databases and published experimental results, a researcher who submits an MIBiG entry is tasked with accurately reporting this information in a Web form, which allows for the data to be stored in a format that facilitates future computational analysis. Relevant information includes publication identifiers associated with the BGC, parameters of the associated genomic loci, information about the chemical compounds(s) encoded, and experimentally verified gene functions. The curation of this information ultimately facilitates computational analyses to connect genes to chemical structures, understand cluster environmental diversity, and facilitate cluster engineering.

### How to thoroughly research a BGC

To properly catalogue a BGC, all the available information about that cluster that is present in the literature must be gathered. However, for many attributes of the MIBiG annotation standard, there may be fields that require information about a cluster that is currently unknown. Therefore, one must first thoroughly search through the literature before submitting a MIBiG entry on a given BGC, to capture all that is known about it.

Peer-reviewed scientific articles can be found using several platforms (e.g., Google Scholar, PubMed, etc.,). First, search the name of the natural product, along with “biosynthetic gene cluster” or “biosynthesis.” After finding a paper that thoroughly reports on the BGC, it is helpful to generate – using a platform such as Google Scholar or Web of Science – a list of more recently published papers that have cited the original paper. This process can provide additional information on the BGC. Taking the time to search through the bibliography of the key authors of papers describing the gene cluster can also be helpful, as these scientists are likely to be experts on the biological pathway in question, yielding a source of additional publications and a potential point of contact. At the university level, Inter-Library Loan programs can be utilized to access journal articles for which a local university might not have the requisite credentials. Additionally, a compilation of review literature defining each of the major biosynthetic pathways that MIBiG focuses on, is referenced in the “MIBiG for Undergraduates” section as an additional resource for students to develop their background knowledge.

### Requesting an MIBIG accession number

To determine if the BGC has already been partially or fully annotated by another researcher, check the MIBiG Repository and sort by main product [2]. It is necessary to verify the species whose genome harbors the cluster, because different species can use different mechanisms to produce similar products. Synonymous cluster and organism names are curated by MIBiG. Compound name synonyms are also captured by the entry fields and organism species names are linked to the NCBI standards as all cluster data is based around genome entries. If a partial entry exists, then view the data for the cluster to determine what information has already been gathered and from what sources. To overwrite the partial entry with a completed entry, follow the workflow as if a new entry is being submitted and use the accession number of the partial entry. Submit the new entry to the MIBiG database with the consolidated data of the new findings along with the previous user’s findings. If there is already a fully completed entry, then the entry can be further developed as more experimental data is generated by the scientific community. This is done by updating specific information with the ‘Update form’ on the MIBiG website.

If it appears that the gene cluster has not yet been assigned an MIBiG accession number after searching the MIBiG Repository for the compound and its corresponding BGC, an accession number should be requested on the MIBiG website [2]. To request an MIBiG accession number, provide contact information (the name, email address, and representing institution of the user), the name of the main chemical compound (s) produced by the gene cluster (e.g., erythromycin), and the accession number to the nucleotide sequence (s) containing the gene cluster (most commonly from GenBank [4]), along with the coordinates of the cluster in these nucleotide entries. If the BGC spans the entire nucleotide entry, coordinates are not necessary (e.g., this GenBank entry [5]).

To find a GenBank entry for a pathway, check a paper that reports on the full BGC, which will likely provide the accession number of the relevant nucleotide sequence file. Additionally, GenBank can be used to search for nucleotide sequence files. For best results, GenBank searches should be concise and more detail should be added to narrow down the results of broad searches; however, adding too many terms can derail the search. Boolean logic (‘AND’ or ‘OR’ terms, as well as brackets to delineate their scope) can be used to make search terms more specific (e.g., “aflatoxin[Title] AND “gene cluster” AND (Aspergillus[ORGN] or Penicillium[ORGN]),”). After finding the file with the desired sequence, be sure to verify the file contains all the genes that are believed to be included in the cluster and that the sequence is taken from the appropriate species.

### Step 1: Cluster and compound information

*This section can be completed in tandem with the corresponding Excel spreadsheet “Offline MIBiG Step 1”* (Additional file 1)*. Each bolded section below corresponds to each field or set of fields present on both the Excel template and the MIBiG Web form for Step 1* [2]*. The following fields are applicable to the cluster and compound (s) being reported. There is a field for comments for elaboration on instances where it was unclear how to proceed filling in a field or where the data provided needs further explanation.*

First, provide the name of the contact for correspondence, academic institution or company name, and a contact email address that will be associated with the MIBiG entry.

#### MIBiG accession number

Record the MIBiG accession number assigned to the BGC.

#### Biosynthetic class

Report as to which biosynthetic class the BGC belongs. The MIBiG standard has focused on six major biosynthetic classes: non-ribosomal peptide, polyketide, ribosomally synthesized and post-translationally modified peptide, terpene, saccharide, and alkaloid. Note that natural products created by all other biosynthetic mechanisms fall under the category “other.” Select all the categories that apply to trigger the correct follow-up questions in the spreadsheet/form. For example, a glycosylated polyketide is classified as both a “polyketide” and a “saccharide.”

#### Key publications

Perform a literature search to develop a list of key publications associated with the gene cluster and molecule. This section should include every paper used to gather information as the entire MIBiG entry is prepared. The number of papers available will be subject to the ‘popularity’ of the cluster, i.e. how many publications have been devoted to it. List the papers by inputting their PubMed IDs (PMIDs, not PMCIDs), separated by commas (e.g., ‘12000953,8843436’). The PubMed Single Citation Matcher can be used to look up a PubMed ID [6]. Only enter numeric characters and commas (no spaces). If a PMID is not available, a DOI can be entered instead, without the designation ‘DOI’ itself (e.g., ‘10.1039/c4sc01927j’). (See the section “How to thoroughly research a cluster” above for additional advice on locating the literature.) In the Excel spreadsheet, record what information this source is providing.

#### Complete BGC sequence

Determine if the BGC has been completely sequenced or if the provided sequence is incomplete. A completed sequence must contain every gene necessary to produce the final molecule. One way to verify if a sequence is complete is by searching the literature for statements such as: “the complete biosynthetic gene cluster was isolated and fully sequenced.”

#### Genomic loci

Specify the sequence of the BGC as deposited in a nucleotide sequence database, such as GenBank, ENA, or DDBJ [4, 7, 8]. Specify multiple loci (ranges clarified by coordinates within a sequence file) if the BGC is expressed over multiple parts of the sequence file or over multiple sequence files. The Excel spreadsheet can support six loci, but the final Web form will allow an unlimited number to be entered. It is suggested that researchers take note of additional data in neighboring empty cells whenever the amount of data they collect exceeds the capacity of the Excel template. Regardless, all data will need to be manually transferred from the Excel worksheets to the MIBiG web forms. Include a start and an end coordinate to indicate the location of the cluster within the sequence file, as well as a nucleotide accession number to identify the file in the database. Additionally, indicate if the sequence file is MIxS-compliant [9]. To determine if a sequence data file has met this metadata standard, the presence of a structured comment at the top of the entry will serve as an indicator [10] (see this example [11]); if unknown, mark this parameter as no. If the pathway is split over multiple clusters, add multiple genomic loci. If the sequence has not been submitted to GenBank, ENA, or DDBJ [4, 7, 8] and has not received an accession number, then the MIBiG entry cannot yet be filled out. Refer to “requesting an accession number” for more information about finding a nucleotide sequence file. Lastly, using the provided options, report the type of experimental evidence that was used to associate the genomic sequence with its proposed function. In the Excel document, only one choice may be selected. However, the Web forms will allow for multiple selections. It is suggested that neighboring empty fields of the Excel template be used to store information when more space is needed to provide sufficient information.

#### Chemical compound

Add information about the major chemical compound (s) produced by the pathway. It is likely that the BGC will produce more than just the main bioactive compound (s), for which the pathway is of interest. While it is most important that the bioactive compound is included in the entry, compounds of lesser importance produced by the pathway should also be included. If a BGC creates multiple very similar but distinct compounds, it is best to consider the significance that minor modifications can have on the overall molecule and to air on the side of inclusion. For example, if three polymer-type compounds are produced and each differs solely by the first monomer, it is more feasible to list these as individual compounds compared to a scenario where 25 highly similar polymer-type compounds are produced. If the scenario is such that it remains unclear how to proceed, contact an MIBiG expert (by emailing mibig@secondarymetabolites.org) for help or be sure to elaborate on the confusion in the appropriate “comments” section of the entry. The Excel spreadsheet can support 12 distinct compounds, but the final Web form will allow an unlimited number to be entered. For more information on the Excel template capacity, review Step 1 under genomic loci.

For each of these compounds, first enter its name.

#### Synonyms

Use the chemical databases (listed next) to find all available synonyms of the compound. In this field, only include “common name” synonyms and avoid using International Union of Pure and Applied Chemistry names or database IDs. Separate these synonyms by commas, without using spaces.

#### Deposited in chemical database

To link the MIBiG entry to the information about the compound available in popular databases, search for records of the compound on PubChem, Chemical Entities of Biological Interest, chEMBL, and ChemSpider [12–15].

If available in PubChem, insert the PubChem ID [12]. Insert numeric characters only (e.g., ‘3081434’,). Type ‘0’ if there is an entry but no ID.

If available in chEBI, insert the chEBI ID [13]. Insert numeric characters only (e.g., ‘42355’ instead of ‘CHEBI:42355’,). Type ‘0’ if there is an entry but no ID.

If available in chEMBL, insert the chEMBL ID [14]. Insert numeric characters only (e.g., ‘273387’ instead of ‘CHEMBL273387’,). Type ‘0’ if there is an entry but no ID.

If available in ChemSpider, insert the ChemSpider ID [15]. Insert numeric characters only (e.g., ‘12041’,). Type ‘0’ if there is an entry but no ID.

#### Compound structure

Use the chemical databases (listed above) to find information on the available structure of the compound. This field is mandatory for all structurally characterized compounds, except for large ones, such as most RiPPs and polysaccharides. Chemical structure should be entered as a Simplified Molecular-Input Line-Entry System string, preferentially isomeric type, in order to include information on stereochemistry [16]. If this data is not available on one of these databases, the information can be easily extracted using software such as ChemDraw, for which most universities have a campus license, or using the (free) PubChem Sketcher [17].

#### Molecular formula

Use the chemical databases (listed above) to find an available molecular formula for the compound. If this data is not available on a database, the information can be easily acquired using software, such as ChemDraw or the PubChem Sketcher [17].

#### Exact molecular mass and ion type

Use the chemical databases (listed above) to find the molecular mass of the compound. If this data is not available on a database, the information can be easily acquired using software, such as ChemDraw or the PubChem Sketcher [17].

If the mass was obtained from unprocessed mass spectrometry data, provide the monoisotopic *m/z* of the molecule for the respective ion type. Use a period as a decimal point, not a comma. If the exact mass was reported in the form of a convoluted ion type (e.g., [M + H]^+^, [M + Na]^+^,), select this from the provided options.

#### Technique (s) used to verify structure

Report nuclear magnetic resonance spectroscopy (NMR), mass spectrometry, X-ray diffraction, chemical derivatization, total synthesis, and/or other experimental results used to verify the structure of the natural product. To find sources, search the name of the molecule using a literature database platform (e.g., Google Scholar, SciFinder, etc.,). Also, the Dictionary of Natural Products as well as the open-access compound databases listed above can be useful resources for finding experimental data. The compound name can be searched along with each verification method (e.g., “lysolipin total synthesis”). Any additional papers found should be included in the “key publications” section above.

#### Molecular activities

Select only experimentally-proven activities from the available options, and choose multiple if appropriate, from the provided list to best describe what is known about the compound. This information is likely to be found in the introduction or discussion sections of papers reporting on the cluster. The Excel spreadsheet can support up to five molecular activities per compound, but the final Web form will allow all potential options to be selected. For more information on the Excel template capacity, review Step 1 under genomic loci.

#### Molecular targets

Enter proteins, RNAs or other (macro) molecules targeted by this compound, as text separated by commas. Enter only experimentally proven targets. Use the common name of the specific target molecule if possible and avoid IUPAC or database identification number terminology. This information is often found in the introduction or discussion sections of papers about the gene cluster.

#### Unusual moieties

Unusual moieties are components of a natural product that are synthesized by means not covered by the NRP/polyketide/RiPP/terpene/saccharide/alkaloid biosynthetic mechanisms. Precursors synthesized by a separate, small, stand-alone synthase, such as 6-methylsalicylic acid, should also be indicated.

When entering this information into the Excel spreadsheet, first enter the number of moieties to be reported. The Excel spreadsheet can support up to five moieties, but the final Web form will allow an unlimited number to be entered. For more information on the Excel template capacity, review Step 1 under genomic loci. For each moiety, use the “chemical moiety” field to select from the prepared options. If the moiety is not on this list, select “other” and indicate the type of moiety in the field labeled “specify moiety.” Next, cite the nucleotide sequence of the subcluster responsible for producing this moiety.^(GEN)^ This ^(GEN)^ notation refers to the MIBiG standard for reporting the identifier of a gene(s) (i.e., the user should enter the locus tag, protein ID, or gene ID (in this order of preference) that can be found for each gene in the GenBank, ENA, or DDBJ file [4, 7, 8] and enter this information exactly as it is specified in the nucleotide sequence file).

#### Operons

Include information about operons only if there are experiments reporting that an operon exists within the cluster. If there is no mention of operons, then this section is not applicable. If there is an operon to report, cite the sequences of the genes that are present in the operon. ^(GEN)^ Additionally, provide the type of experimental evidence used to determine that these genes are clustered in an operon. The Excel spreadsheet can support up to six operons, but the final Web form will allow an unlimited number to be entered. For more information on the Excel template capacity, review Step 1 under genomic loci.

#### NRP-specific parameters

This section is only applicable if the cluster is part of the NRP biosynthetic class. Select the most appropriate NRP subclass from the provided options. In many cases, the option “other” should be used for compounds that do not belong to one of the listed subclasses. For more information on how to determine which subclass is applicable, view the “Table of Parent Molecules for Biosynthetic Subclasses” provided in the Supplemental Information section (Additional file 1).

Based on the structure of the molecule, determine whether it is linear or cyclic. The molecule also counts as cyclic if the molecule is a hybrid and the NRP cyclizes onto another part of the hybrid molecule. Internal cyclizations of an amino acid or cyclizations of two adjacent amino acids or ketide groups do not count to define a cyclic compound: a cyclization should bridge residues that would otherwise not be connected, with at least two other residues being present between them.

To determine if the thioesterase in the nonribosomal peptide synthase gene cluster is type I, type II, or both, look at the relation of the TE to the rest of the assembly line. Type II TEs exist as separate and distinct proteins from the pathway, whereas type I TEs are an integral part of the assembly line and constitute a domain within the larger protein complex. If the TE is type I or of unknown type, identify the TE-encoding genes. ^(GEN)^

Review the types of release/cyclization from the provided options and select based on the characterization of the pathway from the literature [18]. Macrolactamization is a process where a linear peptide undergoes cyclization, resulting in a cyclic amide. Macrolactonization similarly forms a cyclic ester. Additionally, macrothiolactonization by similar means forms a cyclic thioester. NRPS TE domains can utilize hydrolysis for off-loading the thioester-tethered peptides [19]. On the other hand, an example of the reductive release approach in action is the reduction domain using NADPH or NADH to free the peptidyl carrier-protein-bound thioester in the form of an aldehyde, before further reduction to an alcohol [20].

#### Polyketide-specific parameters

This section is only applicable if the cluster is part of the polyketide biosynthetic class. Select the most appropriate polyketide subclass from the provided options. In many cases, the option “other” should be used for compounds that do not belong to one of the listed subclasses. For more information on how to determine which subclass is applicable, view the “Table of Parent Molecules for Biosynthetic Subclasses” provided in the Supplemental Information section (Additional file 1).

Based on the structure of the molecule, determine whether it is linear or cyclic. Also, the molecule counts as cyclic if the molecule is hybrid and the polyketide cyclizes onto another part of the hybrid molecule. Internal cyclizations of an amino acid or cyclizations of two adjacent amino acids or ketide groups do not count to define a cyclic compound: a cyclization should bridge residues that would otherwise not be connected, with at least two other residues being present between them.

Selecting the polyketide synthase subclass yields more specific questions. Additional information about TE types and the release/cyclization methods is detailed at the end of the NRP-specific parameters section.

A modular type I PKS is a large protein complex consisting of sequential modules, each of which completes a successive chain lengthening step and intermediate modification. The various enzymes associated with the PKS are organized into modules, each of which contributes one additional building block to the nascent polyketide. A module is composed of domains, each of which have a defined function and are separated from one another by short spacer sequences. For this synthase subclass, provide the starter unit used, the TE type (choose ‘None’ if it concerns a hybrid pathway and the TE is an integral part of an NRPS module at the end of the assembly-line), and the release/cyclization type. If the TE is type II, both, or other, report the TE encoding genes. ^(GEN)^

An iterative type I PKS is a large protein complex consisting of a set of domains, used in a repetitive cycle of chain elongation. For this synthase subclass, provide the starter unit used, the genes in the cluster encoding nonmodular PKS/ketosynthases, ^(GEN)^ and the number of chain synthesis iterations performed by the iterative PKS module. Additionally, identify the iterative PKS subtype based on reducing capability from the provided options. This reduction is traditionally performed by ketoredutase, dehydratase, and enoyl reductase domains. A non-reducing subtype produces each chain extending unit as a carbonyl, whereas a fully reducing subtype will fully reduce each extending unit. A partially reducing subtype works in between these extremes. Next, provide the iterative PKS cyclization type, TE type, and release/cyclization type. If the TE is type II, both, or other, report the TE encoding genes. ^(GEN)^

A trans-acyltransferase type I PKS is a large protein complex capable of producing a polyketide but depends on a separate protein to function as an AT. For this synthase subclass, provide the starter unit used, the genes in the cluster encoding trans-acyltransferases, ^(GEN)^ TE type (choose ‘None’ if it concerns a hybrid pathway and the TE is an integral part of an NRPS module at the end of the assembly-line), and release/cyclization type. If the TE is type II, both, or other, report the TE encoding genes. ^(GEN)^

An enediyne type I PKS produces a polyketide that is an enediyne. For this synthase subclass, provide the starter unit used, the genes in the cluster encoding nonmodular PKSs/KSs, TE type (choose ‘None’ if it concerns a hybrid pathway and the TE is an integral part of an NRPS module at the end of the assembly-line), and release/cyclization type. If the TE is type II, both, or other, report the TE encoding genes. ^(GEN)^

A type II PKS is an aggregation of small monofunctional proteins that are analogous to the domains of a type I PKS. For this synthase subclass, provide the starter unit used, the genes that encode nonmodular PKSs/KSs, ^(GEN)^ polyketide length, genes in the cluster involved in folding and cyclization of the aromatic polyketide, ^(GEN)^ and TE encoding genes. ^(GEN)^

A type III PKS is most recognizable by its lack of acyl-carrier protein domains. For this synthase subclass, provide the starter unit used, the genes in the cluster encoding nonmodular PKSs/KSs, ^(GEN)^ polyketide length, and TE encoding genes. ^(GEN)^

A polyunsaturated fatty acid synthase or related PKS produces PUFAs or related polyketides. For this synthase subclass, provide the starter unit used, the genes in the cluster encoding nonmodular PKSs/KSs, ^(GEN)^ the nonreductive scaffold-modifying domain in this synthase from the provided options (if applicable), TE type, and release/cyclization type. If the TE is type II, both, or other, report the TE encoding genes. ^(GEN)^

For other PKS, provide the starter unit used and the genes in the cluster encoding nonmodular PKSs/KSs. ^(GEN)^

#### RiPP-specific parameters

This section is only applicable if the cluster is part of the RiPP biosynthetic class. Select the most appropriate RiPP subclass from the provided options. In many cases, the option “other” should be used for compounds that do not belong to one of the listed subclasses. For more information on how to determine which subclass is applicable, view the “Table of Parent Molecules for Biosynthetic Subclasses” provided in the Supplemental Information section (Additional file 1). Additionally, the RiPP subclasses can be studied with more detail, using review articles [21, 22].

Based on the structure of the molecule, determine whether it is linear or cyclic. Internal cyclizations of an amino acid or cyclizations of two adjacent amino acids do not count to define a cyclic compound: a cyclization should bridge residues that would otherwise not be connected.

When recording information about RiPP precursor peptides, the gene ID of the RiPP precursor should be provided. ^(GEN)^ Next, RiPP core peptide amino acid sequence (s) are provided by inputting the sequence as single-AA abbreviations (e.g., ‘ITSISLCTPGCKTGALMGC’). If there are multiple sequences, separate them by commas. Then, record the length in amino acids, including N-terminal signal if present, of the leader peptide. Following this, provide the amino acid sequence (s) of cleavage recognition site (s), if known. If there are multiple, separate them by commas. Next, provide the recognition motif in the leader peptide for the modification peptide (e.g., FNLD for certain lanthipeptides.). Record the peptidase (s) involved in precursor cleavage. ^(GEN)^ Finally, provide information about each crosslink with the final peptide, specifically the first and second amino acids involved in the crosslink and the type of crosslink.

#### Terpene-specific parameters

This section is only applicable if the cluster is part of the terpene biosynthetic class. Select the most appropriate terpene subclass from the provided options. In many cases, the option “other” should be used for compounds that do not belong to one of the listed subclasses. For more information on how to determine which subclass is applicable, view the “Table of Parent Molecules for Biosynthetic Subclasses” provided in the Supplemental Information section (Additional file 1).

Also, select the terpene subclass by number of carbon units: hemiterpene (C5), monoterpene (C10), homoterpene (C11 or C16), sesquiterpene (C15), diterpene (C20), sesterterpene (C25), triterpene (C30), sesquarterpene (C35), tetraterpene (C40), polyterpene (>C40), norisoprenoid (C13), and other. Select the final isoprenoid precursor. Then, provide the genes in the cluster encoding terpene synthases/cyclases [23].^(GEN)^ Finally, provide the genes in the cluster encoding prenyltransferases.^(GEN)^

#### Saccharide-specific parameters

This section is only applicable if the cluster is part of the saccharide biosynthetic class. Select the most appropriate saccharide subclass from the provided options on the MIBiG site. In many cases, the option “other” should be used for compounds that do not belong to one of the listed subclasses. For more information on how to determine which subclass is applicable, view the “Table of Parent Molecules for Biosynthetic Subclasses” provided in the Supplemental Information section (Additional file 1).

Then, provide a reference to the sequence of the gene for each glycosyltransferase (s), enzymes that establish natural glycosidic linkages. ^(GEN)^ Report as to whether the glycosyltransferase has a specificity as pertains to the sugar molecule it interacts with, if known. ^(GEN)^ Also, provide evidence for this claimed specificity, select the strongest evidence of the available choices. Additionally, provide the sequence of the gene subcluster responsible for the biosynthesis of that specific sugar, if the gene(s) is encoded within the gene cluster of this entry. ^(GEN)^ Note that a subcluster may consist of genes dispersed throughout the BGC. A subcluster only needs to be declared once, in case there are multiple glycosyltransferases with the same substrate specificity.

#### Alkaloid-specific parameters

This section is only applicable if the cluster is part of the alkaloid biosynthetic class. Select the most appropriate alkaloid subclass from the provided options. For more information on how to determine which subclass is applicable, view the “Table of Parent Molecules for Biosynthetic Subclasses” provided in the Supplemental Information section (Additional file 1).

#### Parameters specific for other classes

This section is only applicable if part or all of the cluster is not characteristic of the six biosynthetic classes presented above. Select the most appropriate custom biosynthetic class from the provided options, or ‘Other’ if none apply. For more information on how to determine which subclass is applicable, view the “Table of Parent Molecules for Biosynthetic Subclasses” provided in the Supplemental Information section (Additional file 1).

In Part 3 of Step 1, finalize the entry of the gene cluster. Add any final comments that may be necessary for MIBiG staff to know while processing the entry. Additionally, if unpublished data about a gene cluster was entered, check the appropriate box to set an embargo on your data from public showing.

Save and exit the Excel document, as Steps 2 and 3 will be completed using the other provided Excel template.

### Step 2: Gene information

*This section can be completed in tandem with the appropriate Excel spreadsheet “Offline MIBiG Steps 2 and 3,” in the sheet called “Step 2” (*Additional file 1*). Each bolded section below corresponds to each column on the Excel spreadsheet and each field on the MIBiG Web form that is applicable to each gene being reported. In the spreadsheet, as well as in the Web form, each row corresponds to an individual gene. There is a field for comments at the end of each row for elaboration on instances where it was unclear how to proceed filling in a field or where the data provided needs further explanation.*

If you have no tailoring reactions, experimentally verified gene functions, knock-out phenotypes or custom gene names to declare for your cluster, click “skip this step” to move onward to Step 3.

The following information will be entered for each gene in the cluster if available. Annotate each gene that is necessary to make the molecule, as specified in the literature. Note that using the nucleotide sequence file alone to select genes for this step could be problematic, as this file could potentially contain more genes than are necessary to create the desired product. In many of the following categories, the field should be left blank (or as “N/A”, where that is the pre-filled option), when the requested information is either not applicable or not available.

#### MIBiG accession

First, visit the Web form for Step 2 [24]. When a compound name and cluster nucleotide source are used to request an MIBiG accession number, the coordinates and functional annotations of all genes within the gene cluster are automatically retrieved from the GenBank entry. By entering the MIBiG accession number and selecting to pre-fill information, these annotations will appear for that cluster. Copy the pre-filled information from the Web form into the Excel template and check that the data is transferred to the appropriate columns. For the Excel spreadsheet, each gene (or row) for the same gene cluster should have the same MIBiG accession number.

#### Gene/contig accession

The genome or contig accession number refers to the database (often GenBank) accession number for the nucleotide sequence file. If the gene cluster is split up over multiple GenBank files, then provide the GenBank accession number for each gene that will refer to a file containing that gene’s sequence.

#### CDS (coding DNA sequence) in annotation

While looking at the GenBank nucleotide sequence file, under the section “features,” every gene should have a feature next to it called “CDS.” If this feature is present, click yes or TRUE in Excel. Untick this box, or select FALSE in the Excel document, if the gene was not included in the original annotation in the GenBank sequence file and therefore would also not have a CDS feature (e.g., for a small RiPP precursor-encoding gene).

#### Start coordinate

In the GenBank file, each gene is presented as, for example, 1...2364 /gene = “calY”. This notation indicates that for the gene “calY,” the start coordinate is 1.

If there is not pre-filled sequenced-based data for a given gene but the gene is annotated in GenBank with a protein ID, then the start and end coordinates for that gene do not have to be provided. In contrast, if a gene is not annotated in GenBank with a protein ID, then start and end coordinates for that gene are mandatory.

#### End coordinate

In the GenBank file, each gene is presented: for example, 1...2364 /gene = “calY”. This notation indicates that for the gene “calY,” the end coordinate is 2364.

#### Protein ID

This refers to the GenBank accession number for the protein produced by the gene. For each gene with CDS features in the GenBank file, each CDS feature should provide the gene name as “/gene = “calY”” and the protein ID as “/protein_id = “BAP05573.1”.” If this is the case for your entry, enter the protein ID accordingly.

#### Gene ID

This refers to the name of the gene. In the GenBank file, each gene feature should provide the gene name as “/gene = “calY”.” “calY” is the Gene ID. If there is no Gene ID type “No gene ID.”

#### Gene function annotation

This refers to a short description of the gene’s function, often referencing the name of the protein it produces. For each gene with CDS features in the GenBank file, each CDS feature should provide the gene name as “/gene = “calY”” and the gene function annotation as “/product = “CalY”.” If this is the case for your entry, enter the gene function annotation as “CalY”.

#### Gene function category

This field should only be filled in if the function of the gene has been experimentally verified (not just inferred based on homology). The link to the publication that characterizes the gene function should be included in the “publications on this gene” field.

#### Tailoring reaction type

This field is only applicable if “gene function category” has been entered as a tailoring reaction. Select the appropriate option from the provided options.

#### Evidence for function

This field is only applicable if “gene function category” has been filled out. Please select the strongest or most decisive type of evidence. In the Excel document, only one choice may be selected. If multiple types are needed, in the Web form use the exact same wording as in the provided options for each descriptor, but separate them by commas: e.g., “Knock-out, Other in vivo study”.

#### Knockout mutation phenotype

If a knockout study was performed on this gene, a short description of the phenotype should be included. The publication that contains the experiment where this knockout study was performed should be included in the “publications on this gene” field.

#### Publications on this gene

Include publications that provide additional information about this specific gene entry. PubMed ID or DOI of a publication that specifically addresses the function of this gene or the protein it encodes, if any, should be included. If multiple, separate the PMIDs/DOIs by commas.

#### Comments

Provide any additional information that may be necessary for MIBiG staff to know when processing your MIBiG annotation.

### Step 3: NRPS and PKS module information (NRPS and PKS only)

*This section should be completed in tandem with the Excel spreadsheet “Offline MIBiG Steps 2 and 3 Form,” in the sheet called “Step 3” (*Additional file 1*). Each bolded section below corresponds to a column in the Excel spreadsheet and each row refers to a different module being reported. After entering information into the spreadsheet, data can be transferred to the appropriate fields in the MIBiG Web form for PKS and NRPS module information. There is a field for comments at the end of each row for elaboration on instances where it was unclear how to proceed filling in a field or where the data provided needs further explanation.*

If your gene cluster does not contain PKS/NRPS modules, click ‘Skip this step’.

#### MIBiG accession

First, visit the Web form for Step 3 [25], enter the MIBiG accession number, and click to “pre-fill information.” When a compound is submitted to MIBiG to receive an accession number, antiSMASH is used to make predictions about the PKS and NRPS modules and their domain components based on the provided genetic sequence [26]. These predictions are included in the pre-filled information; therefore, these data should be checked carefully and adjusted to match the true situation as described in the literature and verified by experiments. The pre-filled information should be copied into the Excel spreadsheet and be further edited in Excel. Check that the data is transferred to the appropriate columns. For the purpose of the Excel spreadsheet, each module (or row) for the same gene cluster should have the same MIBiG accession number.

#### Module number

Counting starts from 1 at the start of the assembly line, and includes loading modules (which are numbered ‘0’). If a module is split across two genes, re-indicate the same module number for both of the genes, but only include the domains present in that gene. Every module with at least a substrate-selecting domain (adenylation/acyltransferase/Co-A ligase) is included in the count.

For PKS/NRPS hybrids, both PKS and NRPS modules are included in the counting. If this PKS/NRPS module is not part of a main assembly-line for producing this compound or if the PKS/NRPS complex is noncanonical and cannot be described in a linear fashion, please enter ‘x’ as the module number. The same applies for monomodular precursor synthases such as 6-methylsalicylic acid synthase, which may be encoded within larger multi-modular NRPS/PKS-encoding gene clusters.

If there are multiple independent assembly-lines involved in synthesis of the main product, these can be indicated as ‘A1, A2, A3, etc.’ and ‘B1, B2, B3’. Similarly, if the assembly-line branches at a later stage to make multiple products, the shared part can be called e.g. ‘1, 2, 3’ and the split part ‘A4, A5’ and ‘B4, B5’. Up to four parallel assembly-lines are supported (with letters A-D).

#### Protein ID

The protein ID corresponding to the different modules should be included with the pre-filled information.

#### PKS/NRPS

Provide whether this module is a PKS or NRPS module.

#### Skipped/iterated

Indicate if the module is skipped or iterated. Either of these characterizations would be specified in the literature defining the cluster. If the module is not designated as either skipped or iterated, then it is assumed to be neither.

#### Evidence for skipping/iteration

If the module is not skipped or iterated, leave as “N/A”. If applicable, provide evidence of skipping or iteration by choosing from the pre-filled selection.

#### Core domains

Please use standard abbreviations, separated by commas, to include the core domains included in each module: AT, KR, DH, ER, KS, T, CAL, C, A, E. Notably, ACP and PCP domains can both be designated as thiolation domains. Do not include custom modifying domains, but select these in the next column. This information is likely part of the pre-filled information but should be checked for its accuracy and adjusted accordingly with the literature and experimentally verified results.

#### Modifying domain

Enter additional modifying domains present in the module that are not included in the list of core domains in the previous step. In the Excel document, only one choice may be selected. If multiple types are needed, in the Web form use the exact same wording as in the provided options for each descriptor, but separate them by commas (e.g., “Methylation, Oxidation”).

#### Acyltransferase/CAL domain specificity

Report as to whether the AT or CAL domain has a specificity for a particular monomer, if known. In other words, does it selectively interact with one type of monomer.

Enter ‘None’ if no AT-domain is present (e.g., for trans-AT modules) or the AT containing module is skipped. Enter ‘Unknown’ if the substrate specificity is not known. In the Excel document, only one choice may be selected. If the specificity is promiscuous (multiple substrates accepted), in the Web form please enter all specificities on the Web form separated by a forward slash (“ / ”) (e.g., “Malonyl-CoA”).

#### Adenylation domain specificity

Report as to whether the adenylation domain has a specificity for a particular monomer, if known.

Enter ‘None’ if no A-domain present or module is skipped. Enter ‘Unknown’ if specificity not known. In the Excel document, only one choice may be selected. If the specificity is promiscuous (multiple), in the Web form please enter all specificities separated by a forward slash (“ / ”) (e.g., “Alanine/Glycine/Valine”).

#### Evidence for specificity

Choose ‘None’ if substrate specificity is not known or not applicable. Please select the strongest level of evidence. Notably, “structure based inference” differs from “sequence based prediction” in that the former refers to analysis based on the structure of the molecule produced by the module and the latter is used when the amino acid sequence that codes for the module and its domains is used for analysis.

#### KR stereochemistry/activity

Verify the stereochemistry of the ketoreduction by reporting the L or D confirmation of the newly produced hydroxyl group. For more information, please see the cited literature [27].

#### Condensation domain subtype

Select the condensation domain subtype. An LCL domain links two L-amino acids, which is standard [28]. A DCL domain bonds an L-amino acid to a D-amino acid at the end of a peptide chain. A Starter C domain adds a beta-hydroxy-carboxylic acid to the first amino acid. Lastly, a Heterocyclization domain not only creates a peptide linkage but also catalyzes the cyclization of cysteine, serine or threonine. For more information, see the cited literature [28]. Epimerization domains are not counted as condensation domains, but as a separate domain type.

#### Epimerization

Tick this box if selected amino acid is epimerized to a D-enantiomer by an epimerization domain or epimerase. If using the Excel spreadsheet, record “TRUE” or “FALSE.”

#### Comments

Provide any additional information that may be necessary for MIBiG staff to know when processing your MIBiG annotation.

All of the data needed to complete an MIBiG entry should now have been recorded in the Excel spreadsheets and this data can be transferred manually to the Web forms on the MIBiG website for submission to the database [2].

### MIBiG for undergraduate students

There is a growing body of evidence that incorporating original research opportunities into the classroom and teaching laboratory leads to benefits for students, faculty, and the progression of science [29–32]. The annotation of partially annotated BGCs, or those that have yet to be deposited into MIBiG, represents a real research problem that can be incorporated into course-based undergraduate research experiences through student-generated contributions to digital community resources [3]. Indeed, similar annotation and curation projects have been successfully executed in the classroom setting [33, 34]. The completion of an undergraduate course in organic chemistry, a basic understanding of bioinformatics and genetics, and the willingness/ability to “learn as you go” should provide sufficient background knowledge for a student to complete an MIBiG entry. However, there is a foundation of familiarity with the six major classes of natural product biosynthetic pathways that would greatly aid a submitter’s ability to thoroughly research a cluster. As such, we recommend that key learning objectives for a course incorporating MIBiG deposition include the following elements: i) developing a sense of familiarity with biosynthesis and the various biosynthetic classes; and ii) learning to navigate and critically evaluate primary literature. To lower the barrier to meet these key objectives, we collected relevant published review material for students to use as resources as they develop their familiarity with each of the major classes: non-ribosomal peptides [35–39], polyketides [39–43], ribosomally synthesized and post-translationally modified peptides [21, 44, 45], terpenes [23, 46], saccharides [47, 48], and alkaloids [49–52]. Also, a very comprehensive textbook on natural product biosynthesis has recently been published by Walsh and Tang [53] and covers all six major biosynthetic classes. With each successive completed MIBiG entry, it is expected that a student will thoroughly build their skills to explore scientific literature.

To ensure that high-quality data is gathered, we recommend that each BGC be randomly assigned to two students, who will independently annotate the corresponding pathway; any field inconsistent between a pair of submissions can be manually evaluated and refined by the instructor or an experienced researcher. Alternatively, each BGC can be assigned to a single student and the instructor can vet the entry prior to deposition. Student gains from the MIBiG annotation project can be evaluated via the growing body of CURE assessment tools [54] or by using MIBiG-specific student surveys that we provide as Supporting Information. Lastly, students should be informed that accuracy over completeness is a preferred strategy for completing the MIBiG forms. In fact, it is likely that all the information requested by the MIBiG standard has not yet been experimentally determined for a given cluster; therefore, it is recommended that fields be left blank when it seems there is no applicable data in existence. Any concerns can be addressed by contacting an MIBiG expert (mibig@secondarymetabolites.org).

### Supporting information

We provide a number of resources to both facilitate and complement MIBiG data entry. Two Excel documents (“Offline MIBiG Step 1” and “Offline MIBiG Steps 2 and 3”) can be used to organize and store data as it is gathered prior to entry submissions. Additionally, the “Table of Parent Molecules for Biosynthetic Subclasses” is provided to simplify the classification of a cluster/compound into the appropriate biosynthetic subclass. To provide for assistance with overcoming any logistical challenges while completing an entry, we have created a tutorial video that overviews a step-by-step process of gathering the requisite data for one cluster, althiomycin [55] (Additional file 2). Lastly, we provide assessment strategies that can be used alongside the implementation of MIBiG as a pedagogical strategy to help gauge the impact and effectiveness of this research-based learning experience as an educational strategy (pre-activity evaluation [56], post-activity evaluation [57]).


**Additional file 2:** MIBiG entry tutorial video. (MP4 497087 kb)


## Additional files

Additional file 1: Supporting documents for MIBiG entries. (ZIP 1328 kb)

## Abbreviations

A: adenylation (domain)
AT: acyltransferase
BGC: Biosynthetic gene cluster
CAL: Co-A ligase (domain)
CoRe: Contributions to digital community resources
CURE: Course-based undergraduate research experience
DH: dehydratase (domain)
E: epimerization (domain)
ER: enoylreductase (domain)
KR: ketoreductase (domain)
KS: ketosynthase (domain)
MIBiG: Minimum Information about a Biosynthetic Gene cluster
NRP (S): nonribosomal peptide (synthetase)
PKS: polyketide synthase
RiPP: ribosomally synthesized and post-translationally modified peptide
T: thiolation (domain)
TE: thioesterase (domain)
