# Supplementary material for: A standardized workflow for submitting data to the Minimum Information about a Biosynthetic Gene cluster (MIBiG) repository: prospects for research-based educational experiences
Source: Stand Genomic Sci. 2018 Jul 11;13:16. doi: 10.1186/s40793-018-0318-y (PMC6042397; doi:10.1186/s40793-018-0318-y)
Supplement: Supplementary file 2 — Supporting documents for MIBiG entries. (ZIP 1328 kb) [file 40793_2018_318_MOESM1_ESM.zip › Subclass Table .docx]

Table of Parent Molecules for Biosynthetic Subclasses

Non-ribosomal peptides (NRPs)

| Members of the **beta-lactam** class all contain a four-membered cyclic amide. One example, penicillin G, is shown below.   |
| --- |
| **Lipopeptides**, some of which are functionally Ca^+2^-dependent, are peptides with a lipid or saturated carbon chain moiety. One example, surfactin, is shown below. The type of lipid moiety should be reported.   |
| **Cyclic depsipeptides** are cyclic peptide chains that contain both amide and ester linkages in close proximity (often alternating). One example, etamycin, is shown below.  **** |
| **Glycopeptides** are peptides with sugar moieties attached. One example, vancomycin, is shown below.  **** |
| **Glycopeptidolipids** are peptides with both sugar and lipid moieties. One example, myxotyroside B, is shown below.  **** |
| **Siderophores** are known for their ability to chelate Fe^+3^. One example, enterobactin, is shown below. Siderophores can only be assigned based on molecule activity, as this subclass is structurally diverse. However, many NRP siderophores contain an iron-chelating amino acid, 2,3-dihydroxybenzoate.   |
| **Pyrrolobenzodiazapine** have a central pyrrolo[2,1-c][1,4]benzodiazepine core. This class of NRPs has an established role of binding with dsDNA. One example, anthramycin, is shown below.  **** |
| **Uridyl peptides** contain a uridyl moiety, a group formed by loss of a 2'-, 3'- or 5'-hydroxy group from the ribose moiety of uridine (shown below).  **** |

Polyketides

| **Ansamycins** are define by having aliphatic bridge linking two non-adjacent positions of an aromatic component [1]. One example, geldanamycin, is shown below.   |
| --- |
| **Angucyclines** start with a 1,2-Benzanthraquinone (shown below) framework of decaketide origin and feature an alkyl group at C-3 and oxygen functionalities at C-1 and C-8 [2].  **** |
| **Anthracyclines** consist of a fused aromatic ring system and an amino sugar [3]. One example, daunorubicin, is shown below.   |
| **Aryl polyenes** feature an aryl moiety bound to a polyene carboxylic acid, which is sometimes esterified with a dialkylresorcinol system [4]. One example, xanthomonadin I, is shown below.  **** |
| A **benzoisochromanequinones** exhibit three fused rings, consisting of modified benzene, quinone and stereospecific pyran rings [5]. One example, actinorhodin, is shown below and exhibits two of these moieties.   |
| A **chalcone** features a ketone with one aromatic substituent and the other containing an α,β-double bond relative to the carbonyl. One example, chalcone, is shown below.  **** |
| An **enediyne** is a compound containing two triple bonds and one double bond. One example, 4-Dihydroxyethyl-8,9-epoxy-enediyne, is shown below.   |
| **Macrolides** are cyclic compounds made up of a large lactone ring, to which deoxy sugars are often attached. One example, erythromycin, is shown below.   |
| **Polyethers** are a class of compounds with multiple ether groups. One example, brevetoxin A, is shown below.  **** |
| **Tetracycline** compounds are denoted by the presence of a common four ring structure. One example, tetracycline, is shown below.   |
| **Tetracenomycin** polyketides are based on the tetracene structure. One example, tetracenomycin D3, is shown below.  **** |
| **Polyphenols** are characterized by the presence of multiple phenol groups. One example, theaflavin-3'-gallate, is shown below.  **** |
| **Stilbene** polyketides are derivatives of the compound stilbene. One example, resveratrol, is shown below.  **** |

Ribosomally synthesized and post-translationally modified peptides (RiPPs)

| **Lanthipeptides** are peptides that contain a lanthionine (shown below), an analogue of cysteine consisting of two alanine residues connected by a single sulfur atom.   |
| --- |
| **Linaridins** are characterized by *C-*terminal aminovinyl cysteine residues. One example, cypemycin, is shown below.  |
| **Proteusins** are all derived from giant toxic peptides called polytheonamides and undergo massive modification throughout their biosynthesis (usually upwards of 50 steps) [6]. A proteusin cluster contains a characteristic nitrile hydratase leader peptide in the precursor gene [7]. |
| **Linear azole(in)e-containing peptides (LAPs)** contain thiazoles and oxazoles, or their reduced thiazoline (2,3, 2,5, or 4,5) and oxazoline forms. One example, plantazolicin, is shown below.  **** |
| **Cyanobactins** are unique in that they are protected at their *N-* and *C-*termini by macrocyclization [8]. One example, trunkamide, is shown below.   |
| **Thiopeptides** are characterized by a nitrogen-containing six-membered rings (such as piperidine, dehydropiperidine, or pyridine) substituted with multiple thiazole rings and dehydroamino acids (an amino acid usually with a carbon-carbon double bond in its side chain) [9]. One example, lactocillin, is shown below  **** |
| **Bottromycins** contain a unique macrocyclic amidine (a complex where one carbon is used to form an amine and an immine) and rare β-methylated amino acid residues [10]. One example, bottromycin A2, is shown below.  **** |
| **Microcins** are characteristically low molecular weight toxic peptides, produced by bacteria [11]. One example is subtilosin A; see “sactipeptides.” |
| **Lasso peptides** consist of a macrolactam ring, which is comprised of seven to nine residues, and a linear *C-*terminal peptide tail [12]. One example, sungsanpin, is shown below.  **** |
| **Microviridins** are tricyclic depsipeptides (containing amide and ester linkages) known to act as serine proteinase inhibitors [13]. One example, microvidin I, is shown below.  **** |
| **Sactipeptides** contain intramolecular linkages between the sulfur of cysteine residues and the alpha carbon of another residue [14]. One example, subtilosin A, is shown below.   |
| **Head-to-tail cyclized peptides** are cyclized between their *N-* and *C-*terminus and range in size between 30–70 amino acids [7]. |
| **Amatoxins** feature *N-*to-*C* cyclization, as well as a tryptathionine moiety, formed by the crosslinking of cysteine and tryptophan residues [15]. They vary little from their backbone structure. One example, alpha-amanitin, is shown below.   |
| **Phallotoxins** feature *N-*to-*C* cyclization, as well as a tryptathionine moiety, formed by the crosslinking of cysteine and tryptophan residues [15]. One example, phallodin, is shown below.  **** |
| **Cyclotides** are smaller peptides, characterized by their head-to-tail cyclized peptide backbone and their three interlocking disulfide bonds [16]. |
| **Orbitides** are plant-produced cyclic peptides that lack disulfide bonds [7]. |
| **Conopeptides** are small peptides, created as neurotoxins, and exhibit many disulfide bridges [17]. |
| **Glycocins** are bacterially produced toxic peptides, containing sugar moieties. |

Terpenes

| **Sterols** are solid, mostly unsaturated, polycyclic alcohols. One example, cholesterol, is shown below.   |
| --- |
| **Dolichols** are long-chain mostly unsaturated compounds, terminating in an α-saturated isoprenoid group, containing a hydroxyl group. One example, dolichol-14, is shown below.  **** |
| **Hopanoids** are pentacyclic molecules based on the structure hopane (shown below).  **** |
| **Quinones** are derived from aromatic compounds through the conversion of an even number of methine groups to carbonyl groups, with any necessary rearrangement of double bonds, resulting in a fully conjugated cyclic dione structure. One example, p-benzoquinone, is shown below.  **** |
| **Gibberellins** are tetracyclic diterpene acids. One example, Giberellin A12, is shown below.  **** |
| **Carotenoids** are tetraterpenoids and feature a polyene hydrocarbon chain, sometimes terminated by rings, and may have additional oxygen atoms attached. One example, beta-carotene, is shown below.   |
| **Tocopherols** are methylated phenols. One example, alpha-Tocopherol, is shown below.  **** |

Saccharides

| **Hybrid or tailoring saccharide** designation is used to describe compounds that contain a saccharide component in a larger aglycone molecule. One example, noglamycin, is shown below.   |
| --- |
| **Oligosaccharides** are sugar polymers made up of two to ten monomers [18]. One example, raffinose, is shown below.  **** |
| **Aminoglycosides** contain an amino-modified glycoside moiety. One example, kanamycin A, is shown below.  **** |
| **Aminocyclitols** contain either five or six-membered carbon rings, with incorporated amine group substituents. One example, streptomycin, is shown below. Aminocyclitols are found as a component of aminoglycosides.   |
| **Lipopolysaccharides** are large molecules featuring a lipid and a polysaccharide, containing an O-antigen (repetitive glycan polymer) and two cores (oligosaccharides). One example, lipopolysaccharide, is shown below.  **** |
| **Exopolysaccharides** are high molecular weight polymers that generally contain monosaccharides and some non-carbohydrate substituents (such as acetate, pyruvate, succinate, and phosphate). |
| **Capsular polysaccharides** are water-soluble, often acidic, have molecular weights on the order of 100–2000 kDa, are linear, and feature regularly repeating subunits of one to six monosaccharides. |

Alkaloids

| The **pyrrole** subclass is made up of derivatives of its namesake (shown below).   |
| --- |
| The **pyrrolidine** subclass is made up of derivatives of its namesake (shown below).   |
| The **pyrrolizidine** subclass is made up of derivatives of its namesake (shown below).   |
| The **pyridine** subclass is made up of derivatives of its namesake (shown below).   |
| The **piperidine** subclass is made up of derivatives of its namesake (shown below).   |
| The **tropane** subclass is made up of derivatives of its namesake (shown below).   |
| The **quinolone** subclass is made up of derivatives of its namesake (shown below).   |
| The **isoquinolone** subclass is made up of derivatives of its namesake (shown below).   |
| The **aporphorine** subclass is made up of derivatives of its namesake (shown below).   |
| The **quinolizidine** subclass is made up of derivatives of its namesake (shown below).   |
| The **indole/benzopyrrole** subclass is made up of derivatives of its namesake (shown below).   |
| The **indolizidine** subclass is made up of derivatives of its namesake (shown below).   |
| The **imidazole** subclass is made up of derivatives of its namesake (shown below).   |
| The **imidazole** subclass is made up of derivatives of its namesake (shown below).   |
| **Steroidal alkaloids** contain the core steroid structure of seventeen carbon atoms, bound as four connected rings: three cyclohexane rings and one cyclopentane ring. One example, solanidine, is shown below.   |
| **Terpenoid alkaloids** are based on the variously sized terpene skeletons. One example, gentianine, is shown below.  **** |

Other Biosynthetic Subclasses

| **Aminocoumarins** have the core structure of a 3-amino-4,7-dihydroxycumarin ring (shown below) and feature various linkages.   |
| --- |
| **Non-NRP beta-lactams** and **siderophores** are like their NRP counterparts (see above) but are not produced by the same NRP biosynthetic mechanism. |
| The **butyrolactone** subclass is made up of derivatives of its namesake (shown below).  **** |
| The **ectoine** subclass is made up of derivatives of its namesake (shown below).   |
| The **furan** subclass is made up of derivatives of its namesake (shown below).   |
| The **phenazine** subclass is made up of derivatives of its namesake (shown below).   |
| **Homoserine lactones** have a core structure of homoserine (shown below) in its cyclic lactone form.   |
| **Melanins** are produced by the oxidation of tyrosine, followed by polymerization. One example, eumelanin, partially is shown below.  **** |
| **Nucleosides** consist of a nitrogenous base bound to a five-carbon sugar, they can be thought of as nucleotides without a phosphate group.  **** |
| **Phosphoglycolipids** contain a phosphate group, sugar moiety and lipid moiety. One example, glycerylphosphoryldiglucosyl diglyceride, is shown below [19].  **** |
| **Phosphonates** are organic compounds containing phosphorous and feature C−PO(OR)_2_ groups, where R refers to an alkyl group, aryl group, or hydrogen. One example, phosphonic acid, is shown below.  **** |
| **Cyclitols** are cycloalkanes in which at least three atoms of the ring have a hydroxyl group. One example, bornesitol, is shown below.  **** |
| **Glucosinates** feature sulfur and nitrogen and are derived from glucose as well as an amino acid. One example, sinigrin, is shown below.  **** |
| **Amino acid-derived** natural products are made by modification to an amino acid. |
| **Shikimate-derived** natural products are made through the Shikimate pathway, unique to plants and some bacteria. |
| **tRNA-derived** natural products are made through the modification of tRNA [20]. |
| **Fatty acids** are carboxylic acids with long aliphatic chains. One example, myristoleic acid is shown below.   |

References:

1. Rinehart KL, Shield LS. Chemistry of the ansamycin antibiotics. Fortschritte der Chemie Org Naturstoffe. 1976;33:231–307. doi:10.1007/978-3-7091-3262-3_3.

2. Carreño M, Urbano A. Recent advances in the synthesis of angucyclines. Synlett. 2004;2005:1–25. doi:10.1055/s-2004-834813.

3. Hortobágyi GN. Anthracyclines in the treatment of cancer. Drugs. 1997;54:1–7. doi:10.2165/00003495-199700544-00003.

4. Schöner TA, Gassel S, Osawa A, Tobias NJ, Okuno Y, Sakakibara Y, et al. Aryl polyenes, a highly abundant class of bacterial natural products, are functionally related to antioxidative carotenoids. ChemBioChem. 2016;17:247–53. doi:10.1002/cbic.201500474.

5. Lü J, He Q, Huang L, Cai X, Guo W, He J, et al. Accumulation of a bioactive benzoisochromanequinone compound kalafungin by a wild type antitumor-medermycin-producing streptomycete strain. PLoS One. 2015;10:e0117690. doi:10.1371/journal.pone.0117690.

6. Fuchs SW, Lackner G, Morinaka BI, Morishita Y, Asai T, Riniker S, et al. A lanthipeptide-like n-terminal leader region guides peptide epimerization by radical sam epimerases: implications for RiPP evolution. Angew Chemie Int Ed. 2016;55:12330–3. doi:10.1002/anie.201602863.

7. Arnison PG, Bibb MJ, Bierbaum G, Bowers AA, Bugni TS, Bulaj G, et al. Ribosomally synthesized and post-translationally modified peptide natural products: overview and recommendations for a universal nomenclature. Nat Prod Rep. 2013;30:108–60. doi:10.1039/C2NP20085F.

8. Sardar D, Hao Y, Lin Z, Morita M, Nair SK, Schmidt EW. Enzymatic N- and C-protection in cyanobactin RiPP natural products. J Am Chem Soc. 2017;139:2884–7. doi:10.1021/jacs.6b12872.

9. Just-Baringo X, Albericio F, Álvarez M. Thiopeptide antibiotics: retrospective and recent advances. Mar Drugs. 2014;12:317–51. doi:10.3390/md12010317.

10. Crone WJK, Vior NM, Santos-Aberturas J, Schmitz LG, Leeper FJ, Truman AW. Dissecting bottromycin biosynthesis using comparative untargeted metabolomics. Angew Chemie Int Ed. 2016;55:9639–43. doi:10.1002/anie.201604304.

11. Asensio C, Pérez-Díaz JC, Martínez MC, Baquero F. A new family of low molecular weight antibiotics from enterobacteria. Biochem Biophys Res Commun. 1976;69:7–14. doi:10.1016/S0006-291X(76)80264-1.

12. Hegemann JD, Zimmermann M, Xie X, Marahiel MA. Lasso Peptides: An intriguing class of bacterial natural products. Acc Chem Res. 2015;48:1909–19. doi:10.1021/acs.accounts.5b00156.

13. Ahmed MN, Reyna-González E, Schmid B, Wiebach V, Süssmuth RD, Dittmann E, et al. Phylogenomic analysis of the microviridin biosynthetic pathway coupled with targeted chemo-enzymatic synthesis yields potent protease inhibitors. ACS Chem Biol. 2017;12:1538–46. doi:10.1021/acschembio.7b00124.

14. Flühe L, Marahiel MA. Radical S-adenosylmethionine enzyme catalyzed thioether bond formation in sactipeptide biosynthesis. Curr Opin Chem Biol. 2013;17:605–12. doi:10.1016/j.cbpa.2013.06.031.

15. Wieland T, Faulstich H. Amatoxins, phallotoxins, phallolysin, and antamanide: the biologically active components of poisonous Amanita mushrooms. CRC Crit Rev Biochem. 1978;5:185–260. doi:10.3109/10409237809149870.

16. Craik DJ, Daly NL, Bond T, Waine C. Plant cyclotides: A unique family of cyclic and knotted proteins that defines the cyclic cystine knot structural motif. J Mol Biol. 1999;294:1327–36. doi:10.1006/jmbi.1999.3383.

17. Velásquez JE, Van der Donk WA. Genome mining for ribosomally synthesized natural products. Curr Opin Chem Biol. 2011;15:11–21.

18. McCranie EK, Bachmann BO, Goeke K, Schroder W, Lenz J, Piepersberg W, et al. Bioactive oligosaccharide natural products. Nat Prod Rep. 2014;31:1026–42. doi:10.1039/C3NP70128J.

19. Takahashi H, Hayakawa T, Murate M, Greimel P, Nagatsuka Y, Kobayashi T, et al. Phosphatidylglucoside: Its structure, thermal behavior, and domain formation in plasma membranes. Chem Phys Lipids. 2011;165:197–206. doi:10.1016/j.chemphyslip.2011.12.010.

20. Herrmann KM, Weaver LM. The shikimate pathway. Annu Rev Plant Physiol Plant Mol Biol. 1999;50:473–503. doi:10.1146/annurev.arplant.50.1.473.
